# Supplementary material for: Plasma membrane H+-ATPases sustain pollen tube growth and fertilization
Source: Nat Commun. 2020 May 14;11:2395. doi: 10.1038/s41467-020-16253-1 (PMC7224221; doi:10.1038/s41467-020-16253-1)
Supplement: Supplementary file 1 — Supplementary Information [file 41467_2020_16253_MOESM1_ESM.pdf]

Supplementary Information

Plasma membrane H^+-ATPases sustain pollen tube growth and fertilization

Hoffmann *et al.*

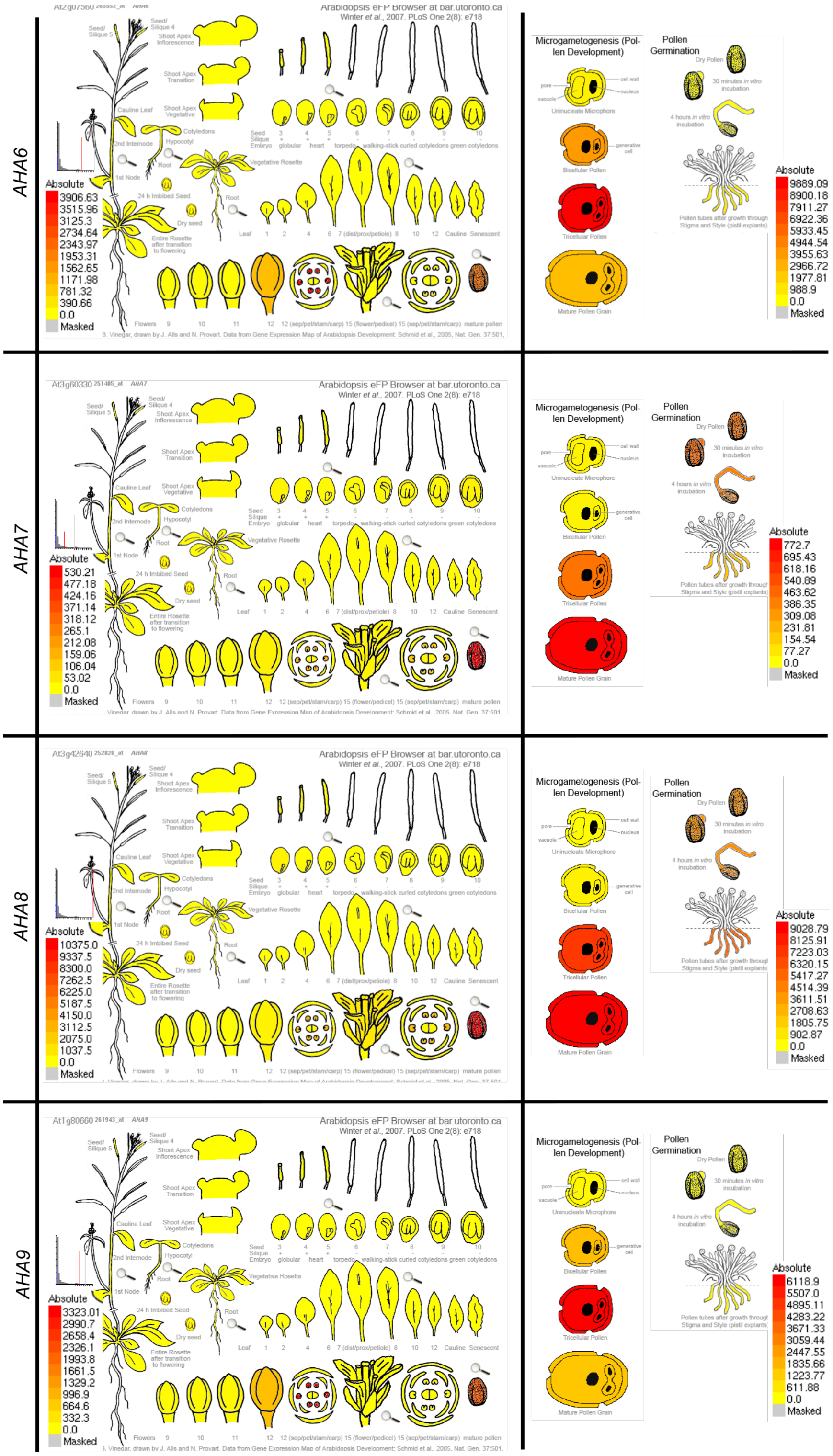

Supplementary Figure 1. Tissue-specific gene expression of *AHA6*, *AHA7*, *AHA8*, and *AH9* in the *Arabidopsis thaliana* Col-0 ecotype. *AHA6* and *AHA9* are expressed during pollen development, *AHA8* in the late stages of pollen development and in pollen tubes, and *AHA7* in pollen grains, pollen tubes, and root hairs (not depicted here) (https://www.bar.utoronto.ca).

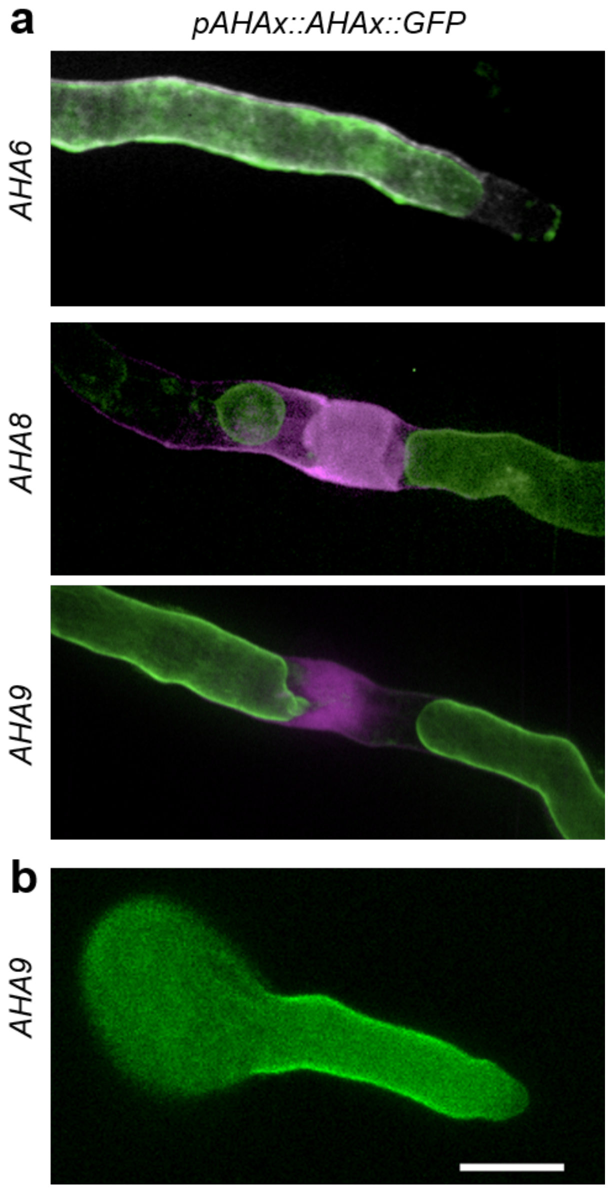

Supplementary Figure 2. Plasma membrane localization of GFP-tagged AHA proteins in pollen tubes. (a) Pollen tubes were plasmolyzed using 70% (w/v) sucrose solution. In AHA6::GFP pollen, the plasma membrane retreated from the tip. In AHA8::GFP and AHA9::GFP pollen, callose plugs (stained with aniline blue, magenta) separated two compartments of the pollen tubes. (b) AHA9::GFP is in the shank region, close to the PT germination site, and is absent from the growing tip. (a,b) Scale bar = 10 µm.

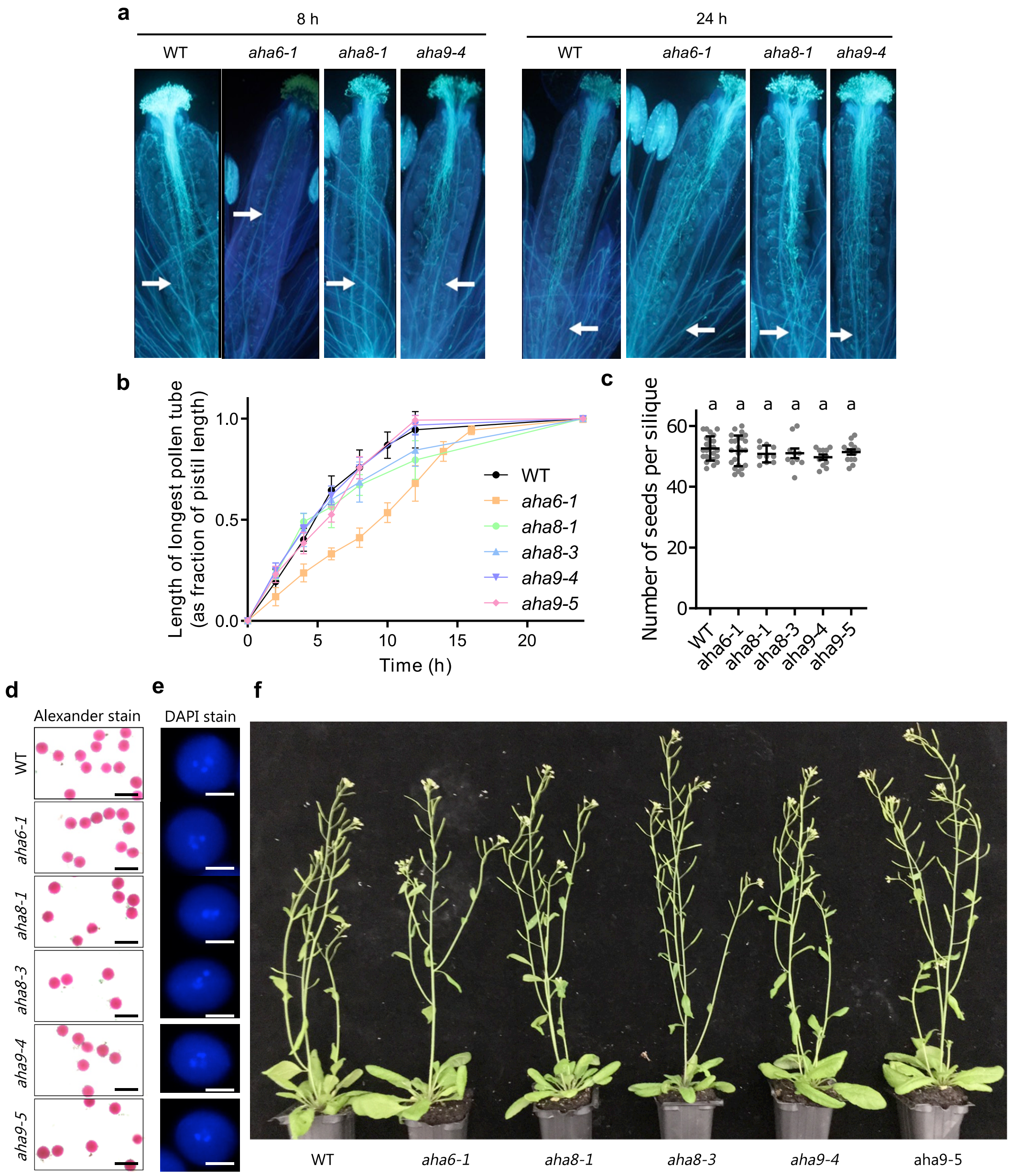

Supplementary Figure 3. Tube growth of *aha6* pollen is slowed down *in vivo*. (a) *In vivo* pollen tube growth visualized by aniline staining. (b) Graph of *in vivo* pollen tube growth. For each data point, at least three *ms1* pistils pollinated with the respective *aha* mutant pollen were scored. Error bars show SD. (c) Seed setting rate of homozygous knockout plants. Seeds of a minimum of twelve siliques were scored per genotype. No significant differences were detected (ANOVA with Bonferroni's Multiple Comparison Test; error bars show SD). (d) Mature pollen stained with Alexander's stain; red dye stains viable pollen grains (scale bar, 50 µm). (e) Mature pollen stained with DAPI; the vegetative nucleus and the two sperm cells are visible (scale bar, 15 µm). (f) Arabidopsis plants do not show aberrant vegetative growth.

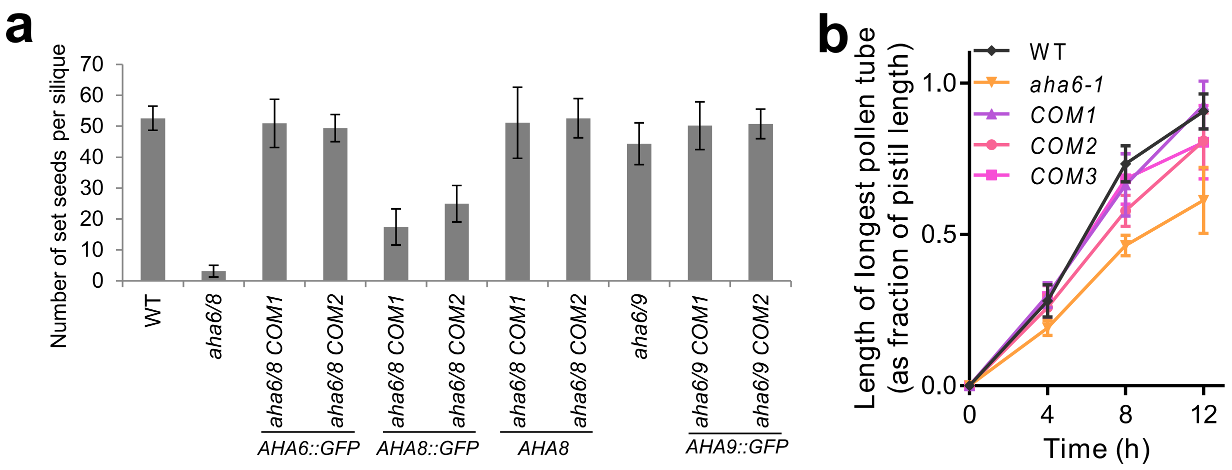

Supplementary Figure 4. *AHA* isoforms functionally complement loss of *aha* genes. (a) *AHA6*, *AHA8*, and *AHA9* functionally complement loss of *aha* genes. The number of seeds per silique in various *aha* double knockout lines and complemented lines was counted. *AHA8::GFP* only partially complemented the reduced seed set phenotype of the *aha6/aha8* line. *AHA8* without the GFP tag was able to fully complement the *aha6/8* phenotype. $n \geq 10$; error bars show SD. (b) *In vivo* pollen tube growth (visualized with aniline blue) of *aha6-1* lines complemented with *pAHA6::AHA6::GFP* show that pollen tube growth rate is similar to that of the WT; error bars show SD.

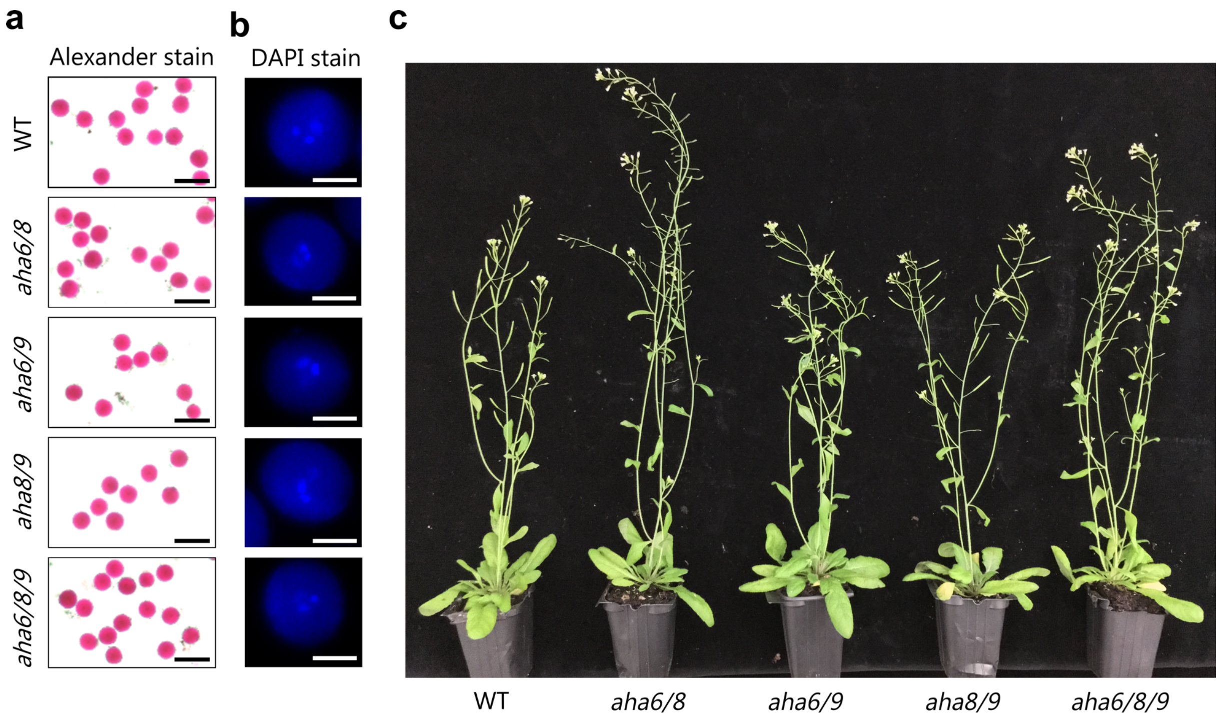

Supplementary Figure 5. Sporophytic and gametophytic analysis. (a) Alexander's stain was used to test pollen viability. Intact pollen grains stain pink (scale bar, 50 µm). (b) DAPI was used to stain the nuclei within mature pollen grains (scale bar, 15 µm). Three nuclei, as seen in the images, indicate normal pollen development. (c) Shoots of mutant lines *aha6/8* and *aha6/8/9,* which develop much less seeds than the other genotypes, grow higher.

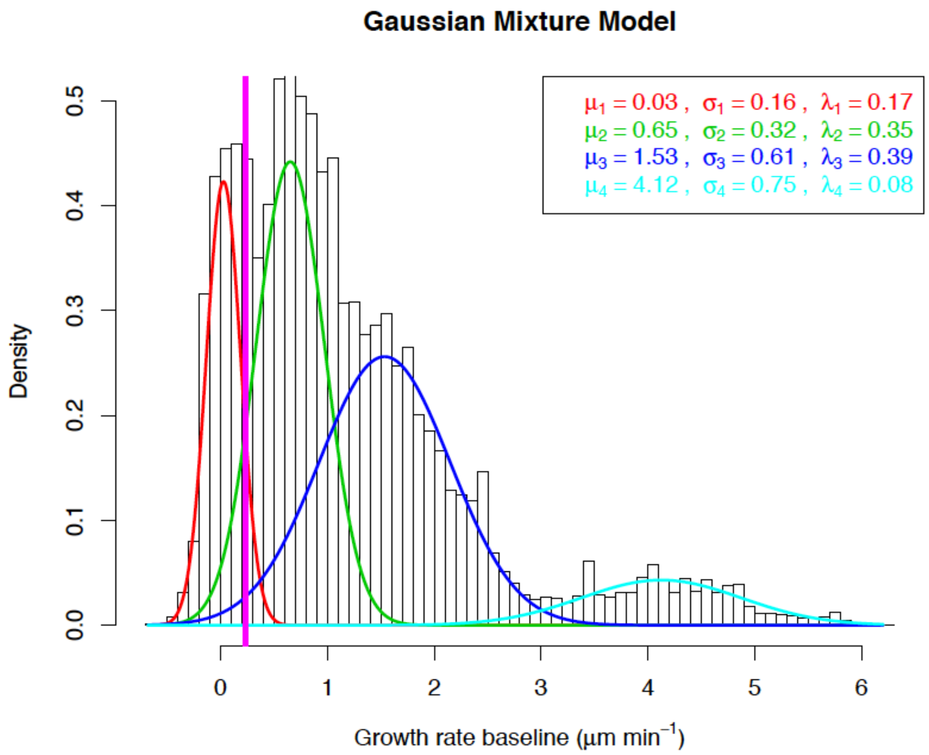

Supplementary Figure 6: **Growth regimes estimated at all time points for all genotypes analyzed**. Probability density of growth rate at any given time, where a mixture of gaussian models was fitted to quantitatively distinguish different growth regimes, yielding four different populational estimates (color curves). Growing and non-growing tubes were distinguished by the intersection between the two distributions with smallest means (magenta line). Raw growth rate estimates were filtered to obtain a growth baseline for every time point as to decrease noise.

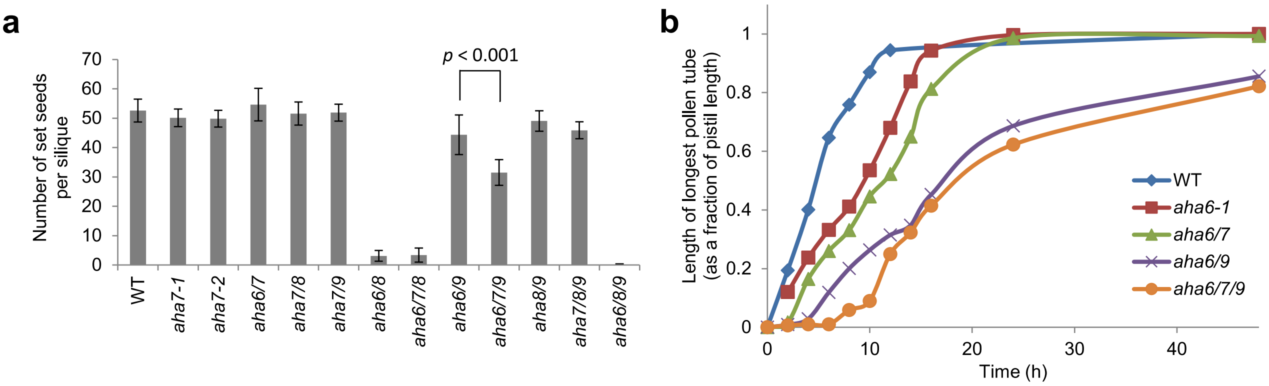

c

Homozygous background	Parental Genotype	Progeny Genotype *AHA/AHA*	*AHA/aha*	*aha/aha*	Ratio	χ^2	*p*
aha6-1	*AHA7/aha7-2*	**59**	**69**	**42**	1.4:1.6:1	**9.42**	**0.009**
aha 8-1	*AHA7/aha7-2*	20	42	24	0.8:1.8:1	0.419	0.8111
aha9-4	*AHA7/aha7-2*	17	47	22	0.8:2.1:1	1.326	0.5154
aha6/8	*AHA7/aha7-2*	21	42	19	1.1:2.2:1	0.60	0.7408
aha6/9	*AHA7/aha7-2*	99	120	35	2.8:3.4:1	**33.02**	**< 0.0001**
aha8/9	*AHA7/aha7-2*	18	42	25	0.7:1.7:1	1.17	0.559
aha6/7/8	*AHA9/aha9-4*	80	70	0	1:0.9:0	**83.46**	**< 0.0001**
aha6/7/9	*AHA8/aha8-1*	210	203	0	1:1:0	**213.68**	**< 0.0001**

d

Female plant	Pollen donor	Progeny phenotype *AHA/AHA*	*AHA/aha*	TE (expected)	χ^2	*p*
WT	*AHA7/aha7-2 (aha6/9)*	124	50	40 (100)	31.471	**< 0.0001**
AHA7/aha7-2 (aha6/9)	WT	83	85	102 (100)	0.024	0.8774

TE (transmission efficiency of gametes) = number of AHA/aha/number of Col-0 x 100.

Supplementary Figure 7. *AHA7* functions in pollen tube growth. (a) The number of seeds per silique in WT and various *aha* lines. Loss of *AHA7* only affects the ability to set seeds when *AHA6* and *AHA9* are lacking as well. n ≥ 10, error bars show SD, unpaired t-test. (b) Pollen tube growth *in vivo*, visualized by aniline blue staining, shows that pollen tube germination is delayed in *aha6/7* (compared to *aha6*) and *aha6/7/9* (compared to *aha6/9*). (c) Offspring distribution analysis confirms that loss of *AHA7* only affects pollen tubes lacking *aha6*, *aha6/9*, or *aha6/8/9* (chi-squared test). (d) Reciprocal crosses with WT showed that loss of *aha7* affects gamete transmission via the pollen tube, but not the ovary (chi-squared test).

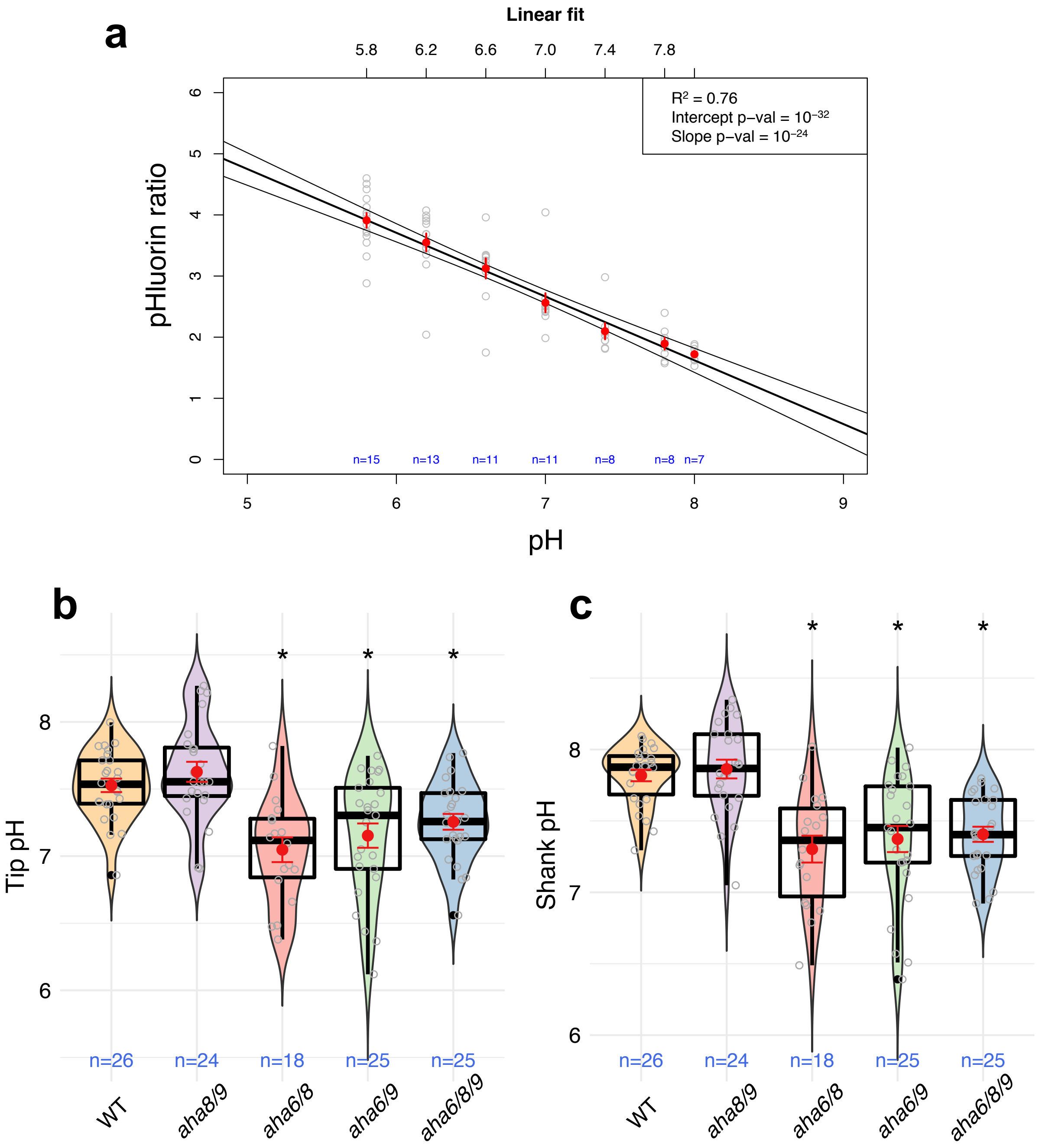

Supplementary Figure 8. Estimates of cytosolic pH based on fluorescence imaging of a genetically encoded reporter. (a) Linear correlation between pHluorin ratio and pH. Ratiometric fluorescence was acquired for tubes treated with the protonophore nigericin and media of different pH. Tip (b) and shank (c) pH estimates (one-way ANOVA comparing to WT, followed by the post-hoc Dunnett test; asterisks mean $p<0.01$). Violin plots show the probability density with color-filled curves obtained from individual observations (open grey circles), with boxplots (thick black lines and outliers as black dots) overlaid with mean and standard error (red circle and lines)

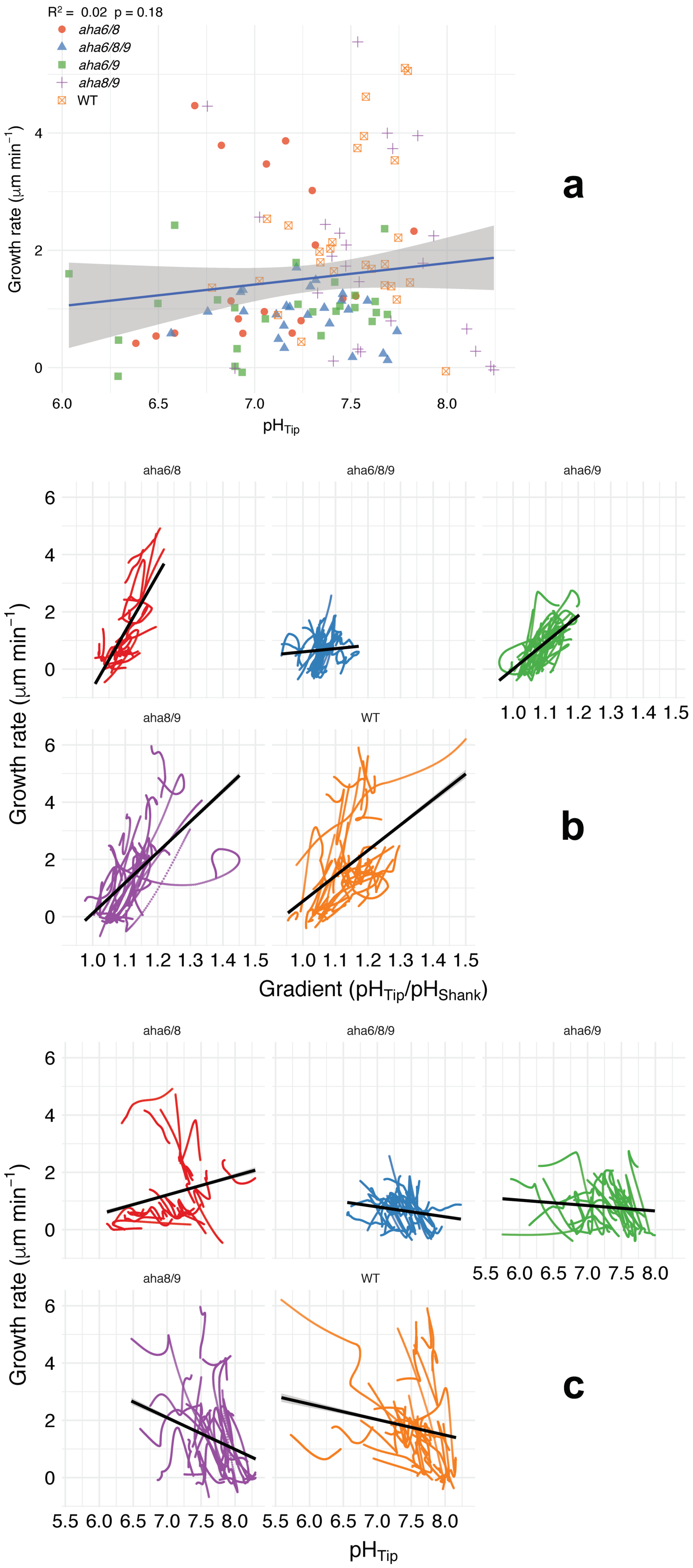

Supplementary Figure 9. Pollen tube growth rate correlates with tip/shank pH gradient but not tip pH alone. (a) The average growth rate and pH at the tip are not correlated, even when all time points under 5 min are considered (b). However, the gradient between tip/shank pH correlates strongly with growth for all time points under 5 min in all genotypes (c), except for the triple mutant, which does not reach sufficiently high growth rates.

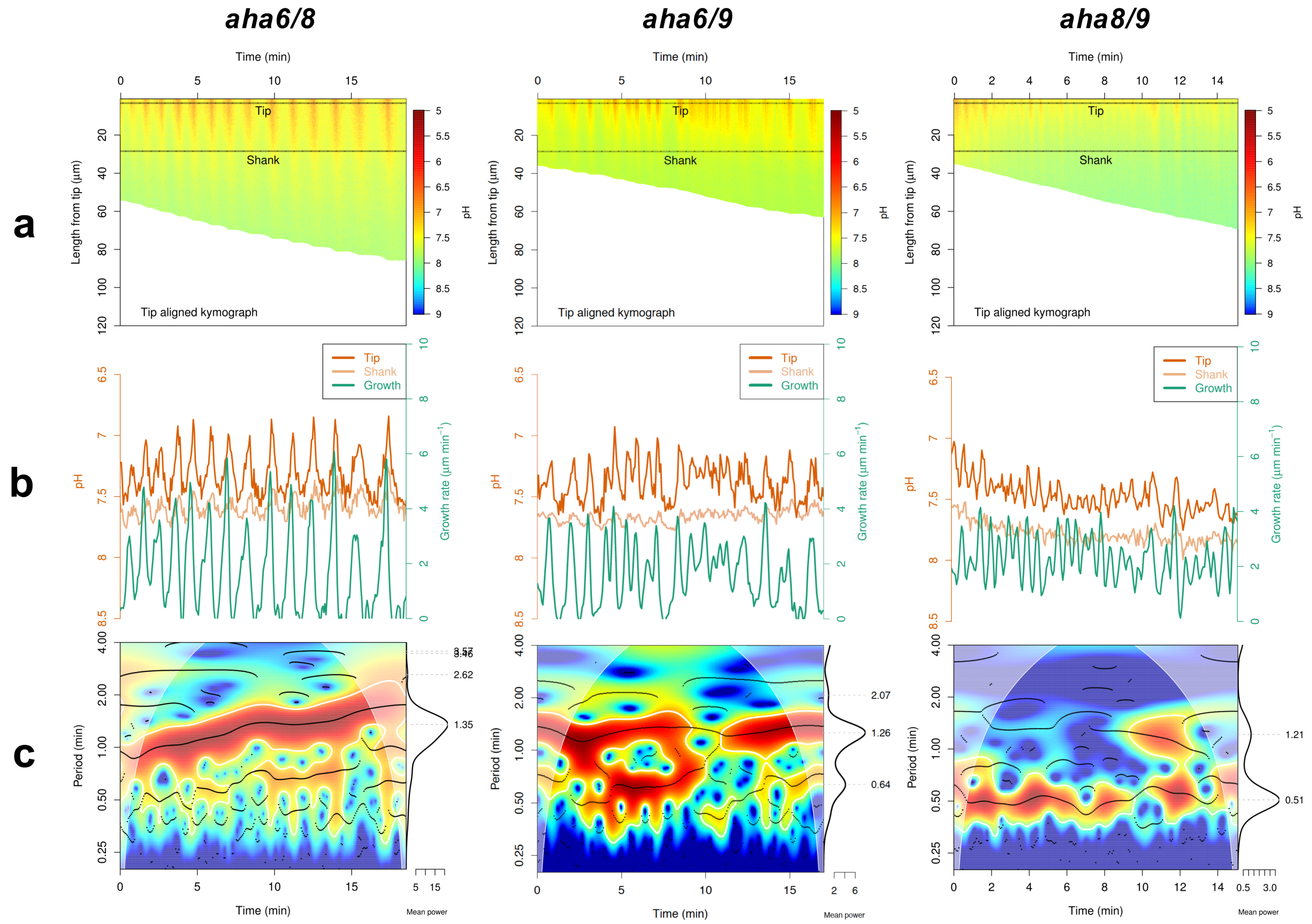

Supplementary Figure 10. Representative time series for remaining genotypes. (a) Tip aligned kymograph with calibrated pH values, with highlighted tip and shank regions used in further analysis; (b) Growth rate (green), cytosolic pH at the tip (orange) and shank (light orange); (c) Synchronization between pH at the tip and growth rate oscillations. Significant joint periodicity across time is shown in the cross-wavelet power spectrum (heat map) demarcated by white lines (p<0.05 compared to an autoregressive process of order 1), where the main instantaneous periods are represented by black lines corresponding to peaks in power (wavelet ridges). The mean power, shown at the right, is averaged across all time points outside the cone of influence (pale region where the estimation is prone to distortion). The overall mean periods present in a series are seen as peaks, being indicated by grey dashed lines and their numerical values.

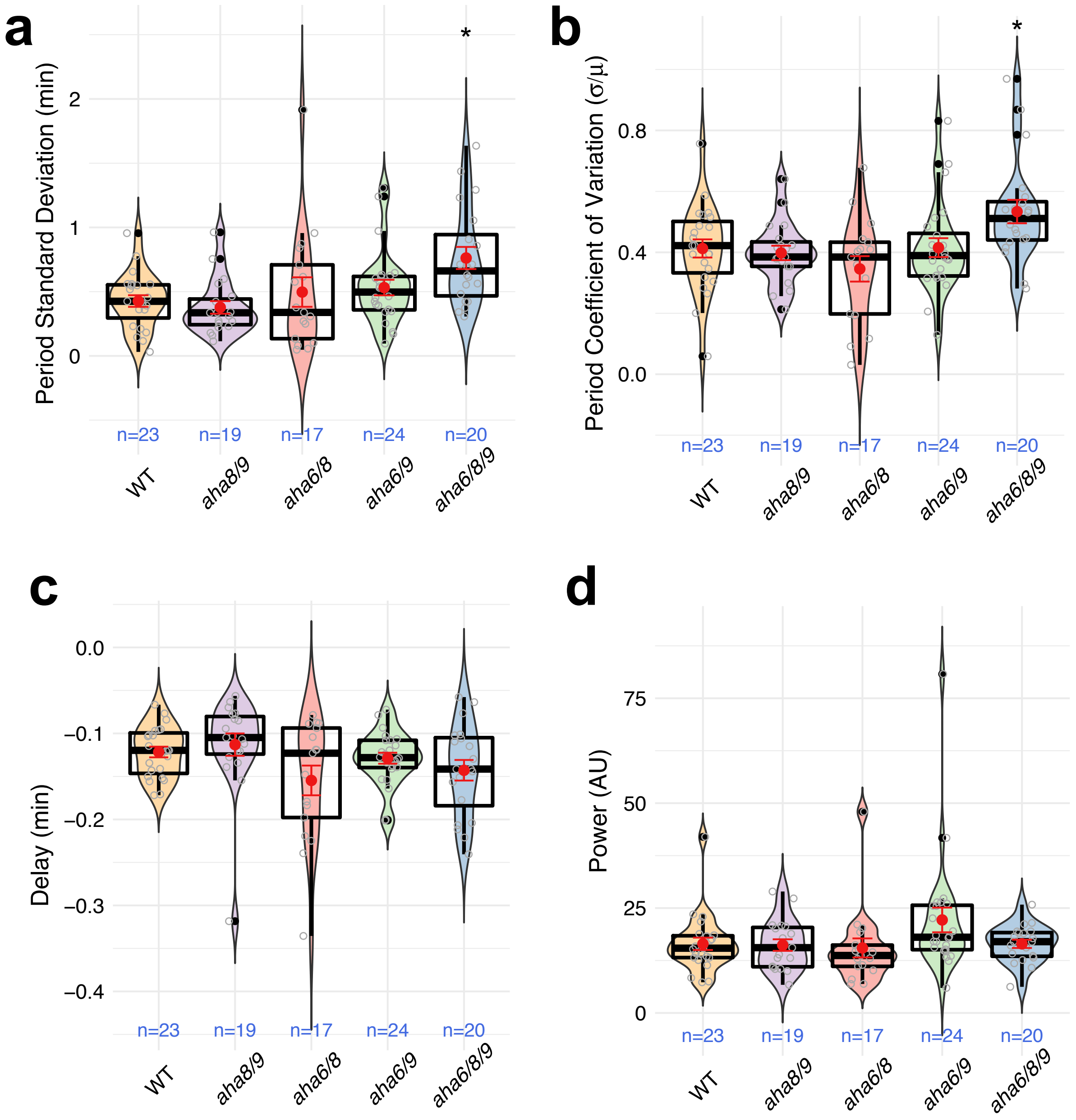

Supplementary Figure 11. Oscillatory characteristics suggesting greater irregularity in critical mutants. The variability in significant periods of synchronized H^+/growth oscillations is shown by its standard deviation (a) and coefficient of variation (b) within series, with highly variable delays in critical mutants between series (c) and no clear difference in amplitude, shown by the median power (d). Violin plots show the probability density with color-filled curves obtained from individual observations (open grey circles), with boxplots (thick black lines and outliers as black dots) overlaid with mean and standard error (red circle and lines). Significant differences with the WT, indicated by asterisks, detected with a one-way ANOVA followed by a nonparametric post-hoc Dunnett test ($p<0.05$).

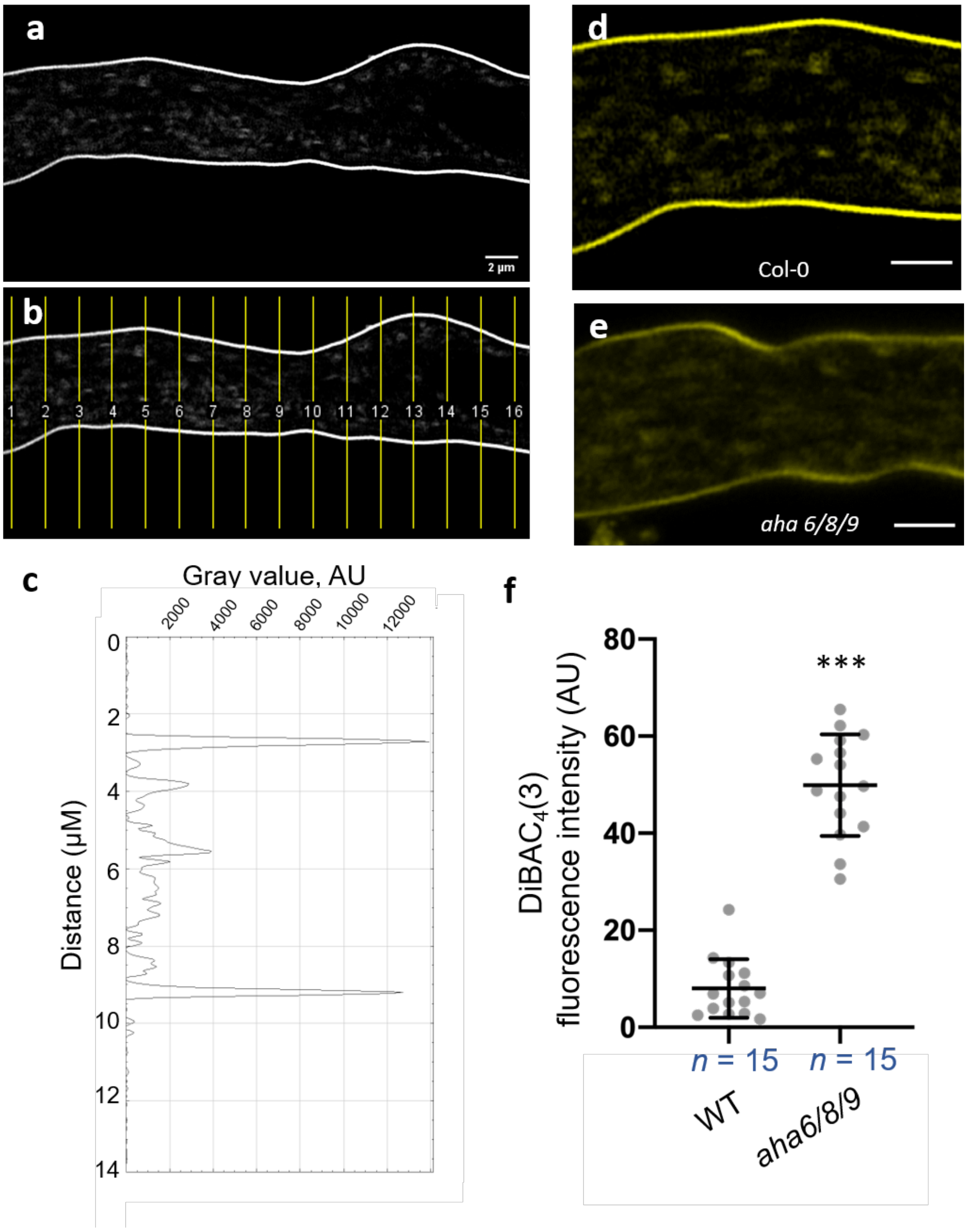

Supplementary Figure 12: The plasma membrane is less negative in *aha 6/8/9* mutant pollen tubes. Images of pollen tube shank segments stained with ANNINE-6-plus were analyzed using ImageJ by drawing transects that were 2 µm apart and perpendicular to the PT's growth axis (a,b). Membrane fluorescence values were averaged for three maxima pixels around the peak value in each transect for both sides of the membrane, left and right (c). Fluorescence intensity was normalized by the background and cytoplasmic fluorescence within each image. Final values are the median of fluorescent intensities between 10–20 µm from the tip (d,e). Scale bars = 2 µm. Control experiments were done by quantifying square regions of interest (ROIs) of 2 × 2 µm of the cytoplasm of the shank region (40–50 µm from the tip). (f) Quantification of DiBAC$_4$(3) fluorescence intensity in the tip and shank of pollen tubes under treatment with wortmannin. Error bars indicate ±SD (n=15) and asterisks indicate values that are statistically different using unpaired t-test (***$p<0.0001$).

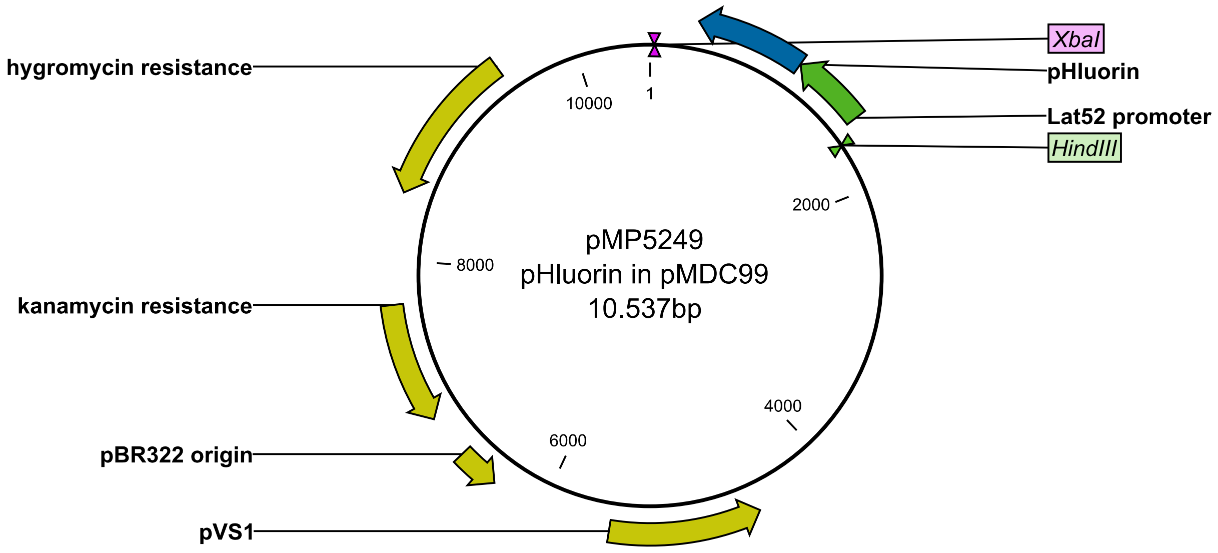

Supplementary Figure 13: Vector map of the pHluorin construct selected for by hygromycin. The pHluorin construct, driven by the Lat52 promoter, is selected for by kanamycin in bacteria, and by hygromycin in plants.

Supplementary Table 1. Offspring of self-pollinated *AHA6/aha6-1* plants were distributed in a non-Mendelian way

Parental genotype	Progeny genotype				χ^2	*P* value
	AHA/AHA	*AHA/aha*	*aha/aha*	Ratio		
AHA6/aha6-1	61	69	33	1:1.1:0.5	**13.45**	**0.0012**
AHA8/aha8-1	38	74	46	1:1.9:1.2	1.44	0.486
AHA8/aha8-3	21	43	21	1:2.0:1.0	0.01	0.994
AHA9/aha9-4	29	63	29	1:2.2:1.0	0.21	0.909
AHA9/aha9-5	63	122	60	1:1.9:1.0	0.08	0.96

Alleles were detected by PCR in T1 seeds. The expected ratio for non-compromised gametes is 1:2:1. Bold face highlights segregation that is significantly different based on chi-squared test.

Supplementary Table 2. Cross pollination with WT plants showed that the reduced inheritance of the *aha* alleles is caused by reduced transmission through the male gametophyte.

Female plant	Pollen donor	Progeny Genotype			χ^2	*P* value
		AHA/AHA	*AHA/aha*	TE % (expected)		
Col-0	*AHA6/aha6-1*	133	56	40 (100)	**31.37**	**< 0.0001**
AHA6/aha6-1	Col-0	47	47	100 (100)	0	1
Col-0	*AHA9/aha9-4 (aha6)*	51	25	49 (100)	8.9	**0.0029**
AHA9/aha9-4 (aha6)	Col-0	41	42	102 (100)	0	0.9
Col-0	*AHA8/aha8-1 (aha6/9)*	57	0	0 (100)	**57**	**< 0.0001**
AHA8/aha8-1 (aha6/9)	Col-0	41	41	100 (100)	0	1

Alleles were detected by PCR in T1 seeds. TE (transmission efficiency of gametes) = number of *AHA/aha*/number of Col-0 x 100. Bold face highlights segregation that is significantly different based on chi-squared test.

Supplementary Table 3. Primers used in this study.

**Name**	**Sequence**	**Observation**
aha6-1_LB	CGTTTATTTCGGCGTGTAGG	*AHA6/aha6-1* genotyping
aha6-1_RP	TGTGCTCTTCTCTTTGTTTCTCC	*AHA6/aha6-1* genotyping
aha6-1_LP	TGCGATTGATACTTCCATTGTT	*AHA6/aha6-1* genotyping
aha7-1_LB	GAAAAGAAAAACCACCCCAGT	*AHA7/aha7-1* genotyping
aha7-1_RP	AGACCACAATACCCACGAAATC	*AHA7/aha7-1* genotyping
aha7-1_LP	AAATGTTCCCGTTGAAGAGGT	*AHA7/aha7-1* genotyping
aha7-2_LB	AAGGGATTTTGCCGATTTC	*AHA7/aha7-2* genotyping
aha7-2_RP	CACCACGGAGAGCATTGA	*AHA7/aha7-2* genotyping
aha7-2_LP	ACCTTCGCTTTAGGGGCTA	*AHA7/aha7-2* genotyping
aha8-1_LB	AAACGTCCGCAATGTGTTATT	*AHA8/aha8-1* genotyping
aha8-1_RP	CTCTCAACAAGGTGCCATCA	*AHA8/aha8-1* genotyping
aha8-1_LP	TTCGTGTGCTTTTCTTTTAGTCTC	*AHA8/aha8-1* genotyping
aha8-3_KO_FW	GTCTACCACCTCCAGAAGCAA	*aha8-3* genotyping
aha8-3_KO_RV	CGGAACCACCATCAAACAG	*aha8-3* genotyping
aha8-3_WT_FW	TACCACCTCCAGAAGCAATG	*AHA8* genotyping
aha8-3_WT_RV	CTCTCTCAGCCTACAAATAAGAAACA	*AHA8* genotyping
aha9-4_LB	CGTCAATTTGTTTACACAGTAGTATAATC	*AHA9/aha9-4* genotyping
aha9-4_RP	GTAAGCCCATTTTCTGAGGAAC	*AHA9/aha9-4* genotyping
aha9-4_LP	TCGAGTAAGTGTATGTGTTGATCTTC	*AHA9/aha9-4* genotyping
aha9-5_LB	TAACTCAACAGCATCAATCACG	*AHA9/aha9-5* genotyping
aha9-5_RP	TTGGAGGGTCAAAGAGAGGT	*AHA9/aha9-5* genotyping
aha9-5_LP	GAGAAGCAAGAGAAGGCATCA	*AHA9/aha9-5* genotyping
AHA6_GFP_FW	CACCGCTTTCCAAAACAATGCCCTTCAGGA	*pAHA6::AHA6::GFP*
AHA6_GFP_RV	GACGGTGTAGTGTTGGTTAAGATTATCAATGTCAAG	*pAHA6::AHA6::GFP*
AHA8_GFP_FW	CACCCTACATGCCACATCTTTGCT	*pAHA8::AHA8::GFP*
AHA8_GFP_RV	TGCAACAGTGTAGTGTTGTTGAATG	*pAHA8::AHA8::GFP*
AHA9_GFP_FW	CACCGAATATACATTAGGAGTCTTGTCTA	*pAHA9::AHA9::GFP*
AHA9_GFP_RV	TGCCAGGGTATAGTGTTGCTG	*pAHA9::AHA9::GFP*
AHA6_GUS_FW	CACCGCTTTCCAAAACAATGCCC	*pAHA6::GUS*
AHA6_GUS_RV	GTCCACCGGAATTTTCTCC	*pAHA6::GUS*
AHA8_GUS_FW	CACCCTACATGCCACATCTTTGCT	*pAHA8::GUS*
AHA8_GUS_RV	CACTTCTTCCACAGGGATCC	*pAHA8::GUS*
AHA9_GUS_FW	CACCGAATATACATTAGGAGTCTTGTCTA	*pAHA9::GUS*
AHA9_GUS_RV	CTCGATCGGTATTTTCTCCTG	*pAHA9::GUS*
AHA8_FW	CACCCTACATGCCACATCTTTGCT	*pAHA8::AHA8*
AHA8_RV	TCAACCCTAATAATCTAAACCAGGA	*pAHA8::AHA8*
AHA6_cDNA_FW	CACCATGGCTGCTGATATCTCATG	Full length cDNA

AHA6_cDNA_RV	TTAGACGGTGTAGTGTTGGTTAA	Full length cDNA
AHA8_cDNA_FW	CACCATGGCGACTGAATTCTCG	Full length cDNA
AHA8_cDNA_RV	TTAAACAGTGTAGTGTTGTTGAATG	Full length cDNA
AHA9_cDNA_FW	CACCATGGCGGGGAATAAAGAT	Full length cDNA
AHA9_cDNA_RV	TCACAGGGTATAGTGTTGCTG	Full length cDNA
AHA6_Δ_cDNA_FW	CACCATGGCTGCTGATATCTCATGGGA	Truncated cDNA
AHA6_Δ_cDNA_RV	AATGCAGTTTAATTTTCAATCATGTTGTTC	Truncated cDNA
AHA8_Δ_cDNA_FW	CACCATGGCGACTGAATTCTCG	Truncated cDNA
AHA8_Δ_cDNA_RV	TTTACTGGTTGATCATGTTATCCCAA	Truncated cDNA
AHA9_Δ_cDNA_FW	CACCATGGCGGGGAATAAAGAT	Truncated cDNA
AHA9_Δ_cDNA_RV	GGTTCAGTTCTCAATGACATTATCC	Truncated cDNA
PP2AA3_RT_FW	GTTGCACACATTCTTCCTGTGATTG	RT-PCR
PP2AA3_RT_RV	CCAGATCCGTCCTAGTTGGCT	RT-PCR
AHA6_RT_FW	AGTTGCTAGGCTAAGAGAAGTG	RT-PCR
AHA6_RT_RV	AACCGAAGGAGAATCCAAACC	RT-PCR
AHA8_RT_FW	TAACAAAAAGAGAGCAAAAACAAGA	RT-PCR
AHA8_RT_RV	ATCTAAACCAGGACCGAGAGA	RT-PCR
AHA9_RT_FW	TGCAAGGTTAAGGGAACGTCA	RT-PCR
AHA9_RT_RV	AGCCACATCCACTGCTAACAA	RT-PCR
C61_F	ATTGCCATTGTGTTGGCAAATGG	CRISPR target AHA6
C61_R	AAACCCATTTGCCAACACAATGG	CRISPR target AHA6
C65_F	ATTGATACAGAAGTTACCAATGG	CRISPR target AHA6
C65_R	AAACCCATTGGTAACTTCTGTAT	CRISPR target AHA6
C82_F	ATTGAAGGTTTGTCCAGCGATGA	CRISPR target AHA8
C82_R	AAACTCATCGCTGGACAAACCTT	CRISPR target AHA8
C85_F	ATTGAGGAGGAAAGGCGCCGGAT	CRISPR target AHA8
C85_R	AAACATCCGGCGCCTTTCCTCCT	CRISPR target AHA8
primer_F	GAATCGAGTTAAAGGGGATCGACTTCAAGGACGATGGAAA CATTC	Introducing silent mutations
primer_R	GAATGTTTCCATCGTCCTTGAAGTCGATCCCCTTTAACTCG ATTC	Introducing silent mutations
